# Challenges experienced by South Africa in attaining Millennium Development Goals 4, 5 and 6

**DOI:** 10.4102/phcfm.v8i2.947

**Published:** 2016-05-06

**Authors:** Fhumulani M. Mulaudzi, Seepaneng S. Phiri, Doriccah M. Peu, Mmamakwa L.S. Mataboge, Nkhensani R. Ngunyulu, Ramadimetja S. Mogale

**Affiliations:** 1 Department of Nursing Science, University of Pretoria, South Africa

## Abstract

**Background:**

Despite progress made by other countries worldwide in achieving Millennium Development Goals (MDGs) 4, 5 and 6, South Africa is experiencing a challenge in attaining positive outcomes for these goals.

**Objective and setting:**

To describe the challenges experienced by South Africa regarding the successful implementation of MDGs 4, 5 and 6.

**Methods:**

An integrative literature review was used to identify and synthesise various streams of literature on the challenges experienced by South Africa in attaining MDGs 4, 5 and 6.

**Results:**

The integrative review revealed the following themes: (1) interventions related to child mortality reduction, (2) implementation of maternal mortality reduction strategies, and (3) identified barriers to zero HIV and TB infections and management.

**Conclusion:**

It is recommended that poverty relief mechanisms be intensified to improve the socio-economic status of women. There is a need for sectoral planning towards maternal health, and training of healthcare workers should emphasise the reduction of maternal deaths. Programmes addressing the reduction of maternal and child mortality rates, HIV, STIs and TB need to be put in place.

## Introduction

The present article aims to identify and describe challenges in the interventions to attain Millennium Developmental Goals (MDGs) 4, 5 and 6 in South Africa. It is necessary to reflect on the challenges pertinent to attaining these three outcomes to guarantee the most effective and sustainable interventions developed for the Sustainable Developmental Goals (SDGs) 2030.

The 21st century was marked by a global commitment to meet the MDG outcomes in 2015. This was a promising endeavour to benefit populations at large as countries were provided with indicators serving as a compass to give strategic direction to service delivery, and that enabled countries to prioritise their goals. Various interventions were adopted by international organisations to support continental- regional- and country-based interventions. Organisations such as the African Union Commission, the United Nations Economic Commission for Africa, and the African Development Bank were applauded for their response to ensure that Africa addressed the challenges that might be barriers to attaining the MDG.^1^ Over the past 5 years, the MDG implementations have shown eminent improvements towards attaining some of the MDG outcomes. However, it is also clear that some countries, including South Africa, still face challenges in achieving the targeted outcomes.^1,2,3^ Currently, South Africa has adopted the National Development Plan (NDP) 2030 vision that incorporates the SDGs as new interventions. It is therefore crucial to examine previous MDG reports to evaluate the challenges experienced during the programme’s implementation. Evaluating these reports will assist in recommending strategies to facilitate attaining the SDGs 2030 within the NDP. Additionally, the information will assist in monitoring and evaluating programmes for the SDGs 2030.

Although all MDGs are interrelated, MDGs 4, 5 and 6 have always been viewed as health-related goals. MDGs1 and 2 address two issues: the eradication of poverty and access to primary education. However, MDGs 1 and 2 have enormous influence on child and maternal mortality as well as on HIV and AIDS. Access to primary education is important as it provides people with writing and reading proficiency that is needed for health literacy. Globally, 90% access to primary education has been achieved; South Africa attained 98%. Within this 98%, more women achieved primary education than men did.

The eradication of poverty is still a global, continental and national problem as it affects access to treatment during ill health, appropriate housing, safe water, food security and sound nutritional status. Factors such as access to safe and secure work remains a problem as many people have low-paid jobs and vulnerable employment is rife.^1^ Accordingly, although a significant decline in poverty levels was noted in the period 1999–2005, South Africa has considerable urbanisation-related poverty problems as most people do not have secure jobs. Meanwhile, other African countries such as Morocco, Egypt, Ghana, Zambia, Cameroon, Cape Verde and Rwanda are indicative of rural poverty. Moreover, in 2010, approximately 1.21 billion people earned < $1.25 a day, which was an improvement on the 1.91 billion in 1990 who received the same amount or less. Education, poverty and gender issues directly affect the prevention of child and maternal morbidity and mortality and HIV and AIDS.^1^

Most programmes in place to address MDGs 4, 5 and 6 are based on reactionary rather than proactive planning. Reactionary planning is about donor-driven programmes–which are not need-based. These donor-driven programmes do not address the problems within the South African context.^4^ Additionally, the programmes lack acknowledgement of the cultural diversity and collaboration amongst Western healthcare workers and traditional health practitioners. Of importance is that the complexities of the current programmes warrant training of healthcare professionals, which poses challenges to the implementation of strategies to attain the MDG 2030 outcomes. Moreover, some HIV and AIDS programmes are vertically designed, not contextualised, and do not acknowledge cultural diversity. Vertical programmes are designed mostly by policymakers with minimal or no consultation with consumers. The programmes respond to an epidemic, are funded by donors, and have to meet specified health outcomes within a specified time. Providers need training, and the implementation of vertical programmes leads to a selective approach that excludes the comprehensive approach promoted in primary health settings.^4^ Even though vertical programmes sometimes attain set health outcomes, they do not respond to determinants rooted in cultural practices, health promotion behaviours, literacy levels, and socio-economic status. Consumer empowerment is almost non-existent.

The progress related to maternal and child health indicates a decline in child and maternal mortality rates. The United Nations’ target for child mortality of 20 deaths per 1000 births has not been attained as some countries’ child mortality is still 60–80 deaths per 1000.^5^ Central to women and child health outcomes is the HIV and AIDS epidemic.^5^ In South Africa, intensification of the interventions to meet MDGs 4 and 5 pertaining to women and child health was adopted through the maternal deaths reports and perinatal meetings. Basic antenatal care (BANC) was also introduced to improve antenatal visits for better outcomes and healthier babies. However, women still visit antenatal clinics late because of cultural beliefs.^6^ For example, culturally, if a pregnant woman visits the clinic early (during the first trimester) she risks the possibility of being ‘bewitched’ and will consequently deliver an abnormal baby.

Global benchmarking and bargaining for cheaper antiretroviral therapy (ART) is another key intervention as most maternal and child deaths are related to HIV and AIDS. This became obvious when ART programmes such as highly active antiretroviral therapy (HAART), prevention of mother-to-child-transmission (PMTCT), nurse-initiated and-managed ART (NIMART), integrated management of childhood illnesses (IMCI) and voluntary medical male circumcision (VMMC) were introduced. All these endeavours concern the reduction of child mortality rates and increased life expectancy.^7^ In addition, funding models to sustain ART, such as the Presidential Emergency Plan for AIDS Relief Funds (PEPFAR), were developed.^8,9^

## Objective

The objective of the present article is to identify and synthesise various streams of literature on the challenges experienced in South Africa in implementing the interventions needed to attain MDGs 4, 5 and 6.

## Research methodology

The integrative literature review method was chosen to identify and synthesise various streams of literature on the progress made by MDGs 4, 5 and 6 as well as the challenges experienced in the healthcare system.^10^ An integrative literature review is a form of research that reviews, critiques and synthesises literature on a topic in an integrated manner.^11^ This method allows the inclusion of diverse methodologies.^12^ This review comprised available and appropriate literature that provided critical discussion and recommendations on the challenges of MDGs 4, 5 and 6 in South Africa. Literature and related documents were searched via the University of Pretoria database, Allied Health Literature, CINAHL, ebscohost and Google.^12^ The main focus was on reviewing MDG country reports for South Africa, the United Nations and Africa. Maternal and child mortality reports and HIV and AIDS international and national progress reports were also reviewed.

Every article and report was selected and validated carefully. The results were interpreted, skimmed and summarised.^13,14^

## Results

The results were guided by the themes identified and described during the integrated literature review. These themes included (1) interventions related to child mortality reduction, (2) implementation of maternal mortality reduction strategies, and (3) identified barriers to the zero HIV and TB infections and management.

### Interventions related to child mortality reduction

In South Africa, the Problem Identification Programme (PIP) identified the main five challenges contributing to child mortality rates as: pregnancy and childbirth problems; newborn illnesses; childhood illnesses; HIV and AIDS; and malnutrition. These contribute to 260 deaths daily, with 75 000 children not living to their fifth birthday.^15^All five main challenges have poverty as the overarching cause. Most child deaths are related to gender disparity, poverty, poor household knowledge of child health, nutrition practices, and poor sanitation.^16^ An estimated 1.5 million child deaths are related to unhygienic conditions, with 88% caused by diarrhoea.^17^ The lack of intersectoral collaboration to modify causes such as poverty, which manifests in earning < $1.25 a day, affects men (10%) less than women (12%) and further contributes to high child mortality rates.^3^

Globally, over 2.6 billion people lack access to flush toilets for safe sanitation. This factor predominantly affected the poor over the period 1995–2008.^16^ Inadequate sanitation such as communal toilets and the bucket system continues to create unhygienic conditions that undermine child survival in South Africa.^18,19^ The provision of sanitation, water and proper housing will reduce diarrhoea and respiratory conditions, which are the causes of about 22% of deaths in under-one-year-olds.^20^

South Africa’s commitment is shown through the introduction of interventions implemented to curtail child mortality rates (e.g. Every Death Counts Report; Saving the Child Report; PIP). Poverty reduction interventions in South Africa include the provision of subsidised housing, electricity, water and sanitation, refuse removal, transportation, and transfer of township housing stock. Currently, 15 million of the population receive social grants including a child care grant.^1,3^ The demand for these services remains high, which signifies underachievement in the reduction of poverty. Since poverty directly affects child mortality, it follows that there is an inability to attain the targets for the reduction of child mortality rates. The above-stated interventions emphasise service delivery and the implementation processes. However, they lack supervisory and monitoring aspects, which affects the health-related MDGs 4, 5 and 6.

Successful implementation of interventions requires training of healthcare workers to acquire knowledge and skills on new interventions. Skillful healthcare workers have the competency needed to boost their morale and confidence. Training opportunities on programmes such as IMCI are intended to provide child guardians and healthcare workers with competencies and skills in child healthcare. However, unequal provision of such training opportunities was indicated in an adverse report in 2006, that only 60% of healthcare workers were trained in IMCI.^21^ Moreover, 70% of households were assessed and empowered on breastfeeding and home-based emergencies related to child care – which meant that 30% of households were not educated in how to detect symptoms in sick children. IMCI focuses on the empowerment of guardians and healthcare workers with information on common symptoms and home-based care while awaiting healthcare workers’ interventions.^21^ In fact, a lack of knowledge can lead to failure to early identification of signs and symptoms of serious illnesses, thus contributing to the inability to curtail child mortality rates.^2,15^

The management of emergencies is another shortcoming, as healthcare workers reported a lack of skills in advanced emergency care for children < 5 years old.^22^ Fifty-three per cent of child mortality results from factors preventable by healthcare providers.^15^ The strategy to improve health consumers’ health literacy on the prevention of child mortality is also significant. This assertion is based on the fact that many children die at home from diseases such as diarrhoea. If mothers were health literate, they would recognise the signs and symptoms of such serious illnesses and take action to manage the identified problems.^3^ Child mortality owing to avoidable causes was one of the factors that led to initiation of the re-engineering of primary healthcare (PHC). This type of initiative is still piloted; it requires healthcare workers to be co-designers together with families of household interventions. Interventions specific to the family will be created in consideration of local available and acceptable resources.^23^

Regarding health-seeking behaviours, it is noted and documented that about 70% of South Africans use traditional healers before considering Western medical care.^24^ However, it is not reported how child healthcare workers and professionals collaborate with traditional healers as the first line of healthcare provision for under-five-year-old children’s preventive, promotive and curative healthcare. With the increasing number of children on ART protocols, a lack of collaboration between these two sectors can lead to challenges such as drug interactions that might lead to child mortality.^25^ The ART programme is one of the vertical programmes implemented to prevent mother-to-child-transmission of HIV and AIDS. The programmes are vertically designed and aimed at reducing morbidity and mortality in children. This vertical programme is more funder responsive than consumer responsive, but it lacks the inclusion of culture, health literacy and social practices common in consumer environments. The lack of collaboration amongst interventions^26^ causes one to negatively affect the other. Therefore a different approach for integrating goals across sectors to promote cost-effectiveness and efficiency is necessary.

A positive outcome relates to an increase in immunisation coverage as parents begin to understand the necessity to have their children immunised. The increase of immunisation coverage was from 68% in 2001 to 98.3% in 2009. Monitoring of the immunisation status of children with every visit to healthcare sectors; enrolment into day-care centres, pre-schooling and schools are some of the reasons for the marked improvement.^3^ Vitamin A supplementation was implemented because a vitamin A deficiency caused 41% of child mortality. Subsequent to Vitamin A supplementation, the diarrhoea incidence amongst children was reduced by approximately 13% – 21%. In addition, deworming of all under-five-year-old children successfully reduced stunted growth.^1,27^

### Inadequate implementation of maternal mortality reduction strategies

The inadequate implementation of maternal mortality reduction strategies was identified as the second theme in the current article. Despite the strategies in place to reduce maternal mortality in developing countries, including South Africa, the level of maternal mortality remains a concern.^1^ Maternal mortality is defined by the World Health Organization (WHO) as the death of a woman while pregnant or within 42 days of termination of pregnancy. In addition, maternal mortality is further defined irrespective of the duration and site of the pregnancy, and from any cause related to or aggravated by the pregnancy or its management, but not from accidental or incidental causes.^28^

According to the 2013 MDG report from Statistics South Africa, one of the leading causes of maternal deaths in developing countries is the complications related to pregnancy and childbirth. Other causes are inadequate promotion of family planning services; inaccessible termination of pregnancy services, resulting in increased numbers of unsafe abortions; and inadequate prevention of STIs, HIV and AIDS are contributory factors towards high maternal mortality rates.^1^

In recognition of the need to reduce maternal mortality in developing countries, various strategies were initiated. The general objective of the Campaign on Accelerated Reduction of Maternal and Child Mortality in Africa (CARMMA) by the African Union Commission, was to accelerate the implementation of key recommendations and strategies to reduce maternal and child mortality through effective advocacy for quality maternal and child healthcare. In addition, strengthening the health system, empowerment and involvement of community partnerships, and collaboration with relevant stake holders were recommended strategies. The 2005 Continental Policy Framework on the Promotion of Sexual and Reproductive Health and Rights in Africa aims at improving the sexual and reproductive health of men and women; as do the Maputo Plan of Action (2006–2010 extended to 2015), and the Abuja Call for Accelerated Action towards Universal Access to HIV and/or AIDS, Tuberculosis, and Malaria services in Africa (2006–2010 extended to 2015). The National Committee on Confidential Enquiries into Maternal Death (NCCEMD) was also established to deal with confidential enquiries into maternal deaths in South Africa and act as a reporting system for maternal deaths. This enquiry was appointed after the declaration of ’maternal death’ as a notifiable condition by the South African government in 1997.^29^

Even with all the strategies and interventions in place, the rate of maternal mortality in developing countries, including South Africa, is still unacceptably high. The maternal mortality rate in developing countries was 230 maternal deaths per 100 000 live births in 2013, and was 14 times higher than that of developed regions which recorded 16 maternal deaths per 100 000 live births in 2013.^29^ Further highlighting the challenges encountered in achieving MDG 5 is the 510 deaths per 100 000 live births in sub-Saharan Africa, making it the region with the highest maternal mortality rate of all developing regions.^29^

The following are some of the challenges that impede the effectiveness of initiated strategies and further contribute to maternal morbidity and mortality:^30^

- large distances between PHC centres and district hospitals, and from district hospitals to regional (referral) hospitals
- inadequate transport facilities
- lack of knowledge and skills to conduct initial assessments and diagnoses
- women living in poverty
- female illiteracy
- teenage pregnancy.

Inadequate utilisation of facilities and available programmes and interventions further exacerbates the inability to achieve desired goals. For example, the WHO recommends a minimum of four antenatal care visits to ensure the well-being of mothers and newborns.^29^ Delayed antenatal bookings contribute to late recognition of complications such as hypertension, proteinuria, haemorrhage, STIs and/or HIV, anaemia and fetal malpresentations (identified and often treated during antenatal care).^30^

In addition to challenges during antenatal care, substandard postnatal care is regarded as one of the main barriers in reducing maternal and child mortality in developing countries, including South Africa. The provision of quality care during the postnatal period may reduce maternal mortality by a further 60% if mothers are educated to use family planning methods effectively and protect themselves from STIs including HIV and AIDS.^31^ Currently there is a grey area between the discharge of postnatal women from the hospitals and home-based care. Postnatal care is rendered at home by grandmothers and traditional birth attendants who are sometimes assisted by traditional health practitioners.^32^ This group of home-based care providers are sometimes not trained, and there is no supervision or support for follow-up visits from midwives, which can result in delayed recognition of complications, leading to increased mother-and-child morbidity and mortality rates.

While it is clear that the South African government has been facing enormous challenges that inhibited its effort to reduce maternal mortality rates by 2015, other indicators are likely to be achieved as indicated in the 2013 MDG report. For example, the proportion of births attended by skilled health personnel is at 100%, the contraceptive prevalence rate is also at 100%, and antenatal care coverage and at least four visits has been achieved with a 100% rate.^1,33^

### Barriers to HIV, STI and TB infection management

South Africa is still experiencing the challenge of addressing the high dual infection rate of HIV and/or AIDS and TB.^34^ The TB and/or HIV co-infection incidence was estimated to be around 540 per 100 000 population in 2004. It remained consistently high over a period of 6 years with a slight increase to 650 per 100 000 population.^34^ A UN general assembly special session (UNGASS) reported in 2012 that the TB and/or HIV co-infection rate in South Africa was 73%. Some of the contributory causes to HIV and/or AIDS and TB co-infection are non-initiation of HIV testing services, lack of human and material resources, and prevailing stigma.

Firstly, HIV testing services are not initiated by healthcare providers while these services are provided outside of TB services.^34^ This is another vertical programme that fails to put the patient’s needs as a priority; on the contrary, statistics are interpreted as a sign of programme success. Secondly, the lack of human and material resources, such as HIV test kits, is a problem and the persistent community stigmatisation of HIV and AIDS and also TB contributes to barriers to the uptake of HIV and/or AIDS counselling and testing (HCT).

The stated challenges to the management of HIV and/or AIDS and TB led to different countries, including South Africa, coming up with innovative ways to address the pandemic. The South Africa government via its national Department of Health has shown much commitment to halt the transmission of HIV and the spread of AIDS and TB through various interventions outlined in the various National Strategic Plans on HIV and AIDS as well as TB.^34^ Programmes such as PMTCT, NIMART, HCT and provider-initiated counselling and testing (PICT) were put in place to achieve the strategic goals.^35^ In addition, an enhanced investment framework for HIV and AIDS which is country specific and serves as a guide to reach zero infection by HIV and curtail the spread of AIDS and TB, was initiated.^36^ The recommended country specific interventions include what is called a comprehensive care plan that advocates for provider-initiated HIV testing and treatment that incorporates early diagnosis and registration of patients who are diagnosed with TB. The comprehensive care plan encourages the commencement of ART for all HIV-infected TB patients irrespective of CD4 cell count.^34^ Having more patients enrolled on NIMART will reduce the viral load and infection rates, and in turn lower the incidence and prevalence rates.

The pronouncement of zero HIV, STI and TB infections management has been associated with numerous factors affecting the reduction of HIV, STI and TB infections. However, several interventions have been implemented to assist in reducing such infections. According to the two goals in the National Strategic Plan of 2012–2016,^37^ the emphasis is on preventing new HIV, STI and TB infections as well as sustaining health and wellness. These efforts were put together to allow institutions to bind themselves to preventing such infections. South African institutions are expected to join hands to seek innovative ways to sustain interventions over the short, medium and long term.^36^ Of further note, although the interventions of the MDGs have been implemented, the need to establish accessible HIV workplace programmes for health workers still exists because these programmes are not accessible at the workplace.^35^

ART remains a promising yet challenging strategy as it brings both positive and negative effects in the lives of people living with HIV and AIDS. The increased number of people on antiretrovirals (ARVs) puts more pressure on governments and their resources, including nurses as initiators of ART in the eastern and southern regions of Africa. It is estimated that the number of people enrolled on ART programmes increased by 625 000 to an estimated 6.3 million in 2012.^38^ The efforts in place were informed by indicators set out in the 2011 Political Declaration on HIV and AIDS adopted by member states in New York. The members came up with predictive objectives, and members pledged that these objectives would be met by 2015.^38^ NIMART was started and increased the access to ART as people were treated and managed by nurses at PHC level. NIMART has been implemented by nurses since 2007 with good effects. However, despite the significant progress made with the programme, people eligible for ART could not access the treatment owing to numerous factors that related to sustainability, price, suppliers and some logistics issues which affected the running of the programme in South Africa.^38^

Although the figures have stabilised, HIV and AIDS still affect many people. Amongst the < 5 age groups, HIV and AIDS deaths were reported to be 33 149, bearing in mind that children have limited access to ART.^39^ Children’s access to ART is less than that of adults for numerous reasons. Children enrolled for ART at the end of 2005 totalled 71 500, while the number had risen to 456 000 by the end of 2010; but this was still less than a quarter of the children eligible for ART. According to the Millennium Development Country Report,^1^ the attrition rate for children was ≥ 20% within 12 months after having enrolled for ART. However, the success of ART in reducing vertical transmission and child mortality is acknowledged, as more pregnant women access HAART programmes. Vertical transmission, specifically mother-to-child, was reduced by almost 60%, making under-five-year-olds healthier.

## Discussion

It is evident from the review of literature and MDG reports that a great deal has been achieved, based on the set targets. Programmes were put in place to address a reduction in maternal and child mortality rates and those of HIV, STIs and TB. It has been documented that, irrespective of numerous interventions implemented globally, including South Africa, similarities and differences exist across countries on child and maternal mortality rates including HIV, STIs and TB management.^37,38,39^ Poverty remains an overarching shortcoming in achieving MDGs 4, 5 and 6 and is more pronounced as a challenge in realising a reduction in child mortality caused by lack of safe water, sanitation and nutrition.^3,20^ Poverty also affects access to care for pregnant women, owing to inadequate transport to clinics or to tertiary hospitals for receiving expert care. It is also clear that the lack of basic education can cause health illiteracy, which affects the understanding of health communications and information, thus affecting the uptake of health services.^30^

Lack of collaboration between traditional health practitioners and allopathic healthcare practitioners is a challenge as the former are viewed by communities as first line caregivers. Ignoring the existence of traditional health practitioners may be detrimental to the proper uptake of healthcare services.^6^ It is evident that these practitioners play a key role in child care, maternal care and the treatment of HIV/AIDS and TB. Donor-funded programmes have helped immensely but they need to be in accord with community needs. Community members and healthcare workers affected by these programmes must be involved from their inception to the end. The involvement of community members will ensure intensified ‘buy-in’ of methods and programmes.^4^

The training of healthcare workers to utilise or implement new initiatives is critical for the success of programmes. For example, it has been established that NIMART is one of the most successful programmes in improving the uptake of ART.^38^ Currently these short programmes are offered by non-governmental organisations. There are no incentives in the form of occupation specific dispensation (OSD) for nurses who offer NIMART. Monitoring and evaluation of programmes and introduction of incentives may encourage more nurses to go for training. It is also critical that continuing education development mechanisms be put in place to enhance the implementation of programmes.

It is very clear from reports that community members must be empowered to take the initiative for their own health. Intensification of health literacy can assist women during pregnancy, birth and childcare. Health literacy will assist people living with HIV and AIDS and TB to take charge of their own health.

## Conclusion and recommendations

It is recommended that poverty relief mechanisms be intensified as they have a bearing on healthcare and access to proper education. The socio-economic status of women should be improved because it affects maternal health if women are not empowered to sustain themselves. PHC and district-level interventions to reduce maternal deaths owing to haemorrhage should be improved. Poverty remains an overarching obstacle to achieving MDGs 4, 5 and 6, and is more pronounced as a challenge in realising a reduction in child mortality owing to inadequate safe water, sanitation and nutrition. It has a financial impact on already impoverished pregnant women, and inadequate transport poses problems in going to clinics and tertiary hospitals for expert care.

Sectoral planning towards maternal health will reduce barriers, such as improving transport, integration of health services, and women’s health literacy.^30^ Also, women should be encouraged to make use of antenatal care or, if they deliver at home, delivery should be under the supervision of skilled birth attendants who must refer them to health services should the need arise^30^ Pregnant women who are HIV positive must be put on ART programmes and contraception to delay pregnancy or prevent unwanted pregnancies.

More strategies to improve quality care during antenatal and intrapartum care to reduce maternal mortality should be put in place. Medical and nursing training schools should be encouraged to focus on programmes such as the reduction of maternal deaths from obstetric haemorrhage and resuscitation of mother and baby.^3^ Community healthcare workers should educate pregnant women about obstetric haemorrhage, as this is one of the leading causes of maternal deaths.^40^

The literature review and MDG reports provided important evidence that much has been achieved, based on the set targets. Programmes were put in place to reduce maternal and child mortality rates, HIV, STIs and TB. Investigation of the literature also revealed that, irrespective of numerous interventions implemented globally, including South Africa, similarities and differences in child and maternal mortality rates including HIV, STI and TB management have been clearly documented across the countries involved.
